# The role of women in Brazilian ethnobiology: challenges and perspectives

**DOI:** 10.1186/s13002-019-0322-3

**Published:** 2019-08-28

**Authors:** Taline Cristina da Silva, Patrícia Muniz de Medeiros, Natalia Hanazaki, Viviane Stern da Fonseca-Kruel, Juliane Souza Luiz Hora, Stephanie Gomes de Medeiros

**Affiliations:** 1 Universidade Estadual de Alagoas, Maceió, Brazil; 2 Universidade Federal de Alagoas, Maceió, Brazil; 3 Universidade Federal de Santa Catarina, Florianópolis, Brazil; 4 Instituto de Pesquisa Jardim Botânico do Rio de Janeiro, Rio de Janeiro, Brazil; 5 Universidade Federal Rural de Pernambuco, Recife, Brazil; 6 Universidade Federal de Pernambuco, Recife, Brazil

**Keywords:** Women in science, Ethnobiology, Gender bias, Sexism, Social influences

## Abstract

**Background:**

The article aims to analyze the representativeness of women in ethnobiological publications within the Brazilian context, as well as to relate the difficulties faced by women in their scientific careers in terms of gender bias. Biases found in publications are relevant themes to different areas of knowledge, considering the historical persistence of male privilege in these activities. We analyzed the role of women in ethnobiological scientific publications and sought to reflect on gender issues in academic practices and fieldwork.

**Methods:**

We conducted a 28-year survey of academic publications in Brazil, through the Scopus and Web of Science databases, in order to infer the female representation in ethnobiological literature. We also sent 77 questionnaires to ethnobiologists associated with the Brazilian society of ethnobiology and ethnoecology or indicted by associates through snowball sampling.

**Results:**

We observed that there are more articles where the senior author is male (*p* < 0.05). However, there are no differences in the number of publications led by men and women (*p* > 0.05), which shows a positive trend in terms of representation. Within subareas, ethnozoology had more male authors than other subareas of ethnobiology. Articles whose senior authors are men tend to be published in journals with a higher impact (*p* < 0.05). The interviews with Brazilian ethnobiologists showed that 53.2% of the interviewees reported feeling discriminated against in the academic environment because they were women. Moreover, 61.0% said they had disadvantages in collecting data because they were women. Additionally, most of the researchers reported having witnessed cases of sexism in the studied communities.

**Conclusion:**

In the current scenarios of female participation, it is possible to reflect and identify advances and challenges associated with gender bias in ethnobiological studies conducted by Brazilian, both in the emic and etic spheres of research and in our scientific practice. As researchers in the area, we deal directly with social problems in the studied communities, such as violence against women, sexism, and prejudice, as well as the many problems faced in the academic universe itself.

## Introduction

Modern science is not only permeated by sociocultural bias, but it is also effectively made by people, for people, and it reproduces a symbolic apparatus of its society, which is made up of power relations. The choice of a research object, the perspective of the research object, the language with which the object is described, how the results are gathered and described are all imbued with historical and social values.

There is a historical persistence that privileges the male gender; the role of women in science and gender bias can influence several steps of research, from data collection to scientific publication, with relevant topics in all areas of knowledge. Therefore, it is necessary to investigate the current scenarios of female participation in scientific research in order to identify the advances and challenges of such participation, since some areas of knowledge have a deficit in female representation, such as mathematics, engineering, and technology [1].

The origin of male prevalence in modern science stems from various sociocultural contexts, including Western societies, where women had distinct social roles apart from men (see, for example, [2, 3]). For example, caring for the home and children was a unique and exclusive role assigned to women for a long period in history in some societies [4], especially in Western societies where the mainstream academic science originated [5].

These role differences are the result of social constructions. Beauvoir ([6] [1949], p.10) was a pioneer in stating that the differences found between men and women are conceptions created collectively from perceived anatomical differences between the sexes. In the author’s words, “no biological, psychic, or economic destiny defines the figure that the human female takes on in society; it is civilization as a whole that elaborates this intermediary product [...]”.

The inclusion of women in the labor market, especially in the field of science, has been disproportionate compared to men in many countries [5, 7–9]. Although the theme “Gender and Science” has been discussed for more than three decades, women are still underrepresented in disciplines and careers, especially in the areas of science, technology, engineering, and mathematics [10]. In a study conducted in the USA, it was found that the average salary of female scientists is lower than that of men and a disproportionate fraction of women abandon their scientific career in the early stages [11]. Perhaps the shortage of women in science can be explained by socio-cultural factors, such as the difficulty in managing and balancing a career and family or discrimination and harassment, which has been reported by women scientists in large-scale studies conducted in the USA [12] and Great Britain [7].

In Brazil, some metrics have been positive regarding female performance in scientific production. A recent report showed that Brazil (along with Portugal) is among the countries included in the study with the highest female representation in terms of scientific research, with women accounting for 49% of researchers in the country [13]. However, according to the same report, there are still a number of challenges for female inclusion in the field of science in Brazil, since, in the hard sciences, female participation is still much lower than male participation [13]. Additionally, for some areas of knowledge, even when publishing a similar amount of articles to men, women are less likely, for example, to receive scholarships [14].

Ethnobiology studies the different dimensions of the relationship between people and nature [15] and is an integrative area for allying different worldviews and forms of knowledge. However, although integrative, this science can bring challenges in terms of gender relations, since it often requires an immersion in sociocultural systems, usually based on a patriarchal logic. Thus, the collection of ethnobiological data can be skewed by the presence of gender taboos and biases—when men from local communities prefer to talk to male researchers about certain subjects or vice versa [16]—and because dominant, abusive, and aggressive relationships among men over women can be commonly seen in certain settings.

Consequently, in ethnobiology, it is necessary to understand if the integrative nature of this science extends to the questions related to the gender in different stages of this science. Brazil is an excellent scenario for accessing the female role in ethnobiological research, as it leads the scientific production in this area, together with countries such as Mexico and Argentina [17, 18]. Thus, through the scientific context in Brazil, this work aimed to investigate the role of women in ethnobiological scientific publications and to access the primary challenges faced daily by these women in the academic field and in their scientific practices.

## Methods

Data collection took place in two stages. Initially, we investigated the role of Brazilian women scientists in scientific publications in the area of ethnobiology through a systematic review. In a second step, we used semi-structured questionnaires answered by female researchers to access the main challenges faced daily by these women in the academic field and in their scientific practices.

### Bibliographic survey

In order to carry out this first stage of the study, we searched for articles in the Scopus (www.scopus.com) and Web of Science (www.isiknowledge.com) databases, using combinations of the following keywords: *ethnobiology* or *ethnoecology* or *ethnozoology* or *ethnopharmacology* or *ethnobiological* or *ethnobotanical* or *ethnobotany* or *ethnopharmacological* or *ethnoveterinary.* All keywords were combined with Brazil.

We could not access articles prior to 1989 since they did not have online abstracts. Thus, our survey was restricted to the most recent studies published between 1989 and 2017. During the search for articles, we included papers that directly investigated the relationship of human groups with different types of resources in Brazil, thus excluding literature reviews and articles presenting only pharmacological, phytochemical, and bromatological data.

After selecting the articles, the following information was extracted for the compilation of a database: gender of the main author; gender of the senior author; year of publication; number of citations; and area of concentration (ethnozoology, ethnobotany, ethnopharmacology, ethnoveterinary, ethnoecology, ethnobiology, or ethnomedicine). The senior author, in this case, was the corresponding author. The journal impact factor was determined according to data provided by Journal Impact Factors (https://www.annualreviews.org) in 2018.

### Semi-structured questionnaire

For the second stage of the study, we conducted a survey of all the women associated with the SBEE (Brazilian Society of Ethnobiology and Ethnoecology), contacting them at their respective e-mails and asking for their collaboration answering an online semi-structured questionnaire. The questionnaire contained questions about sexism in academic settings and field activities faced by researchers, as well as other situations related to the female gender (Bropriating, Manterrupting, Mansplaining, and Gaslighting, whose definitions are available in Table 1). At the end of the questionnaire, the researchers were encouraged to indicate other researchers to participate, according to the snowball sampling procedure [19]. Participation was voluntary, after free and informed consent.

**Table 1:** Situations associated with gender bias in the professional field

Situation	Concept
Bropriating	When, in the presence of a group, a woman gives an idea that is not heard or considered, and this idea is later repeated by a man and from then on, it is taken into consideration
Manterrupting	When a woman is constantly interrupted by men, unable to present or conclude her idea
Mansplaining	When a woman feels underestimated by a man who tries to explain something she has already shown that she has knowledge
Gaslighting	When a man tries to make a woman think that her reactions are exaggerated or that she is crazy

#### Data analysis

A chi-square test of goodness of fit was used to test whether there are differences between men and women in the number of publications (a) as first authors and (b) senior authors. A chi-square in a contingency table was also used to identify differences in ethnobiology subareas regarding female and male participation. The Mann-Whitney test showed differences in the impact factor according to data provided by Journal Impact Factors of both male- and female-authored publications.

Regarding the answers about sexism faced by the researchers and experiences in the communities, as well as other questions related to the female gender and the practice of ethnobiological research, the data were analyzed with descriptive statistics. For open-ended responses, we constructed, through a discourse analysis of the collective subject [20], the participants’ discourse related to situations of sexism or discrimination due to gender in ethnobiology/ethnoecology fieldwork. These situations occurred during fieldwork, where the researcher felt impaired or disadvantaged for being a woman and in situations that the researcher witnessed sexism suffered by women in the communities/groups with whom she worked. Thus, the key expressions of each response were extracted, followed by the grouping of similar central ideas to construct the collective discourse for each response.

## Results and discussions

### Bibliographic survey

A total of 412 studies were found between 1989 and 2017; there were 217 studies where the first author was male and 195 where the first author was female. There was no significant difference between the two groups (*p* > 0.05). However, there are more articles whose senior author is male 278 (*p* < 0.05), demonstrating a predominance of men leading ethnobiology research groups in Brazil. In regards to the subareas, ethnozoology had a higher concentration of male authors (both first authors and senior authors) (*p* < 0.05). This is most likely because within some ethnozoology approaches, such as hunting, the sample group is composed mainly by the male gender [21, 22]. For women, it may be difficult to establish a relationship of trust with the participant, since some of the methodological procedures in ethnozoology are to follow hunting events, and the presence of women is not always allowed in these practices. From the 89 ethnozoology articles found in the survey, 20 were about hunting and 19 about fishing. It is also worth investigating the participation of female researchers in fishing studies, since it differs from hunting but it is still predominantly performed by men. Impact factors were not significantly different between men- and women-led publications; however, articles whose senior authors were men tended to be published in journals of higher impact (*p* < 0.05), once again demonstrating the historical persistence of privilege for men in research activities. However, this scenario can be changed, as women have excelled in ethnobiology in different Latin American countries [23–25].

### Semi-structured questionnaire

**Fig. 1:**
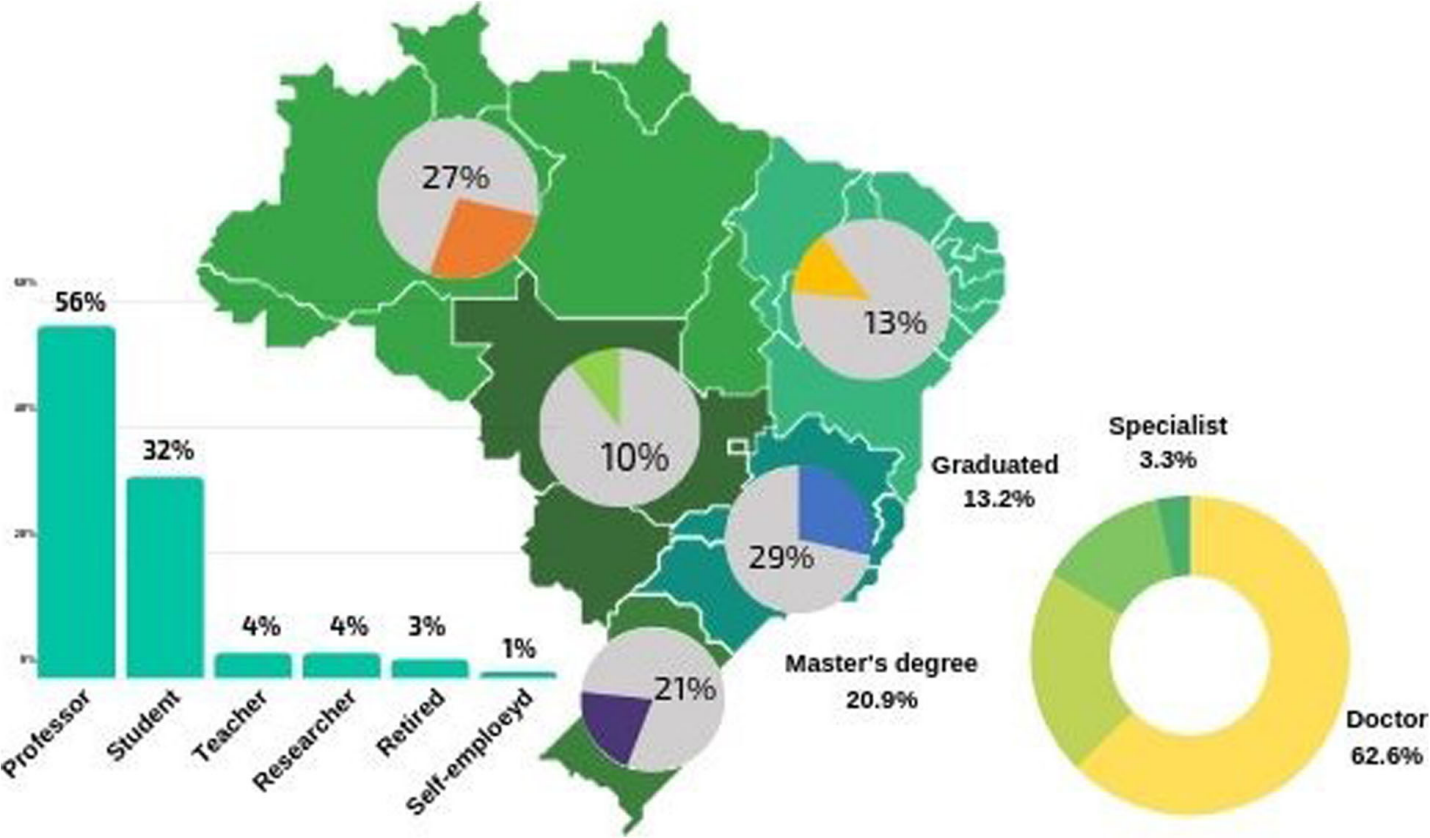
Socioeconomic characteristics of the research participants (*n* = 77 female ethnobiologists), distributed among the five Brazilian regions

A total of 77 women ethnobiologists (researchers), between the ages of 25 and 65, participated in this phase of the research. Most of the participants were professionals from universities or research centers (60.0%), with doctoral education (62.6%), and distributed in 18 Brazilian states (Fig. 1).

**Fig. 2:**
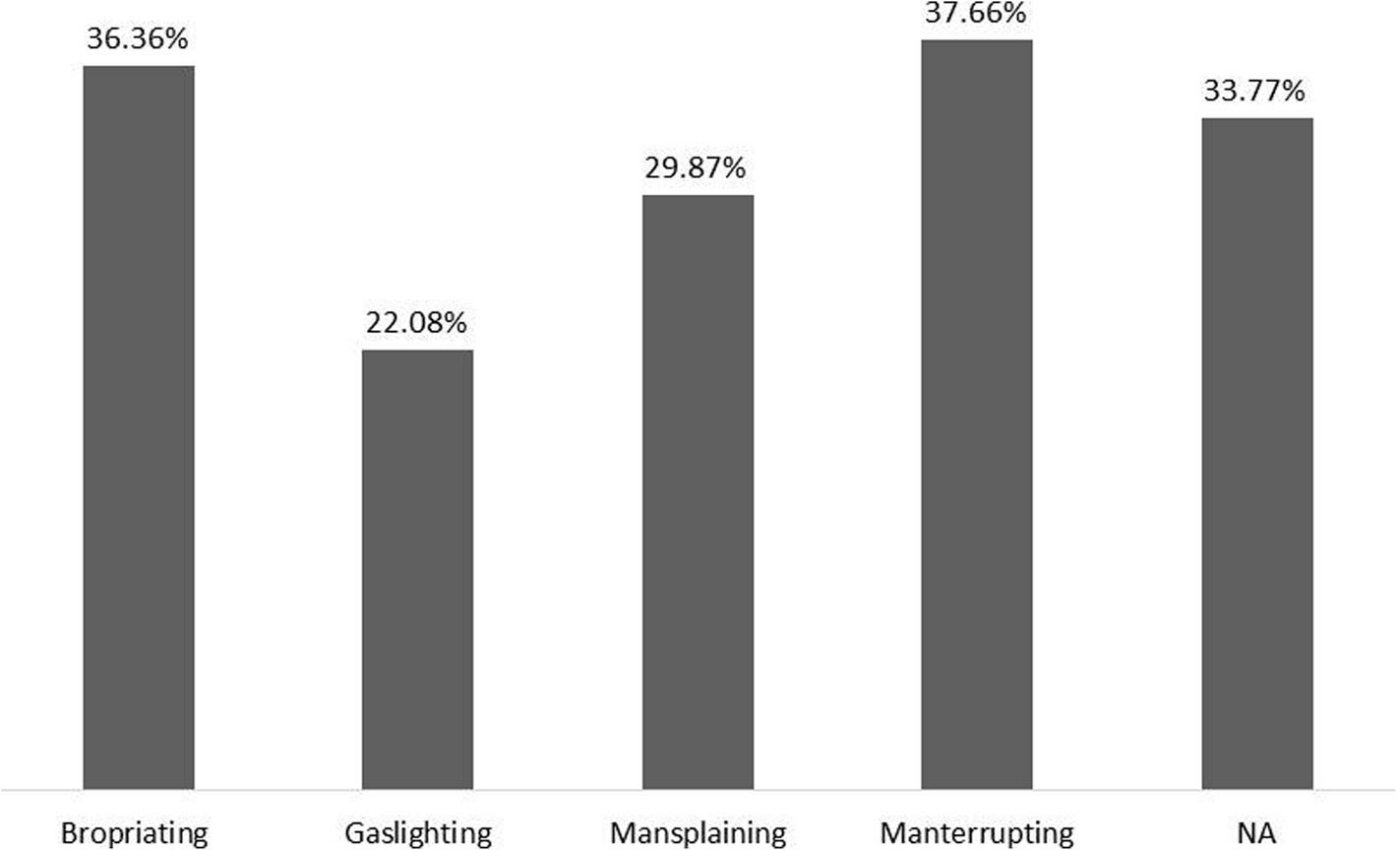
Situations related to gender faced by Brazilian ethnobiologists in the academic environment (*n* = 77 female ethnobiologists)

Most of the participants (53.2%) said they felt discriminated against in the academic environment because they were women. However, when they were confronted with specific situations, there was no consensus. Among these, *Manterrupting* was predominant among respondents (37.7% of interviews), followed by *Bropriating* (36.4%), *Mansplaining* (29.9%), and *Gaslighting* (22.1%) (Fig. 2). When the information on a specific situation was gathered, 58.4% of the women reported having experienced at least one of the situations described above. Interestingly, this percentage is higher than that of women who felt discriminated against in the academic environment. It is possible, therefore, that some women do not see *Manterrupting*, *Bropriating*, *Mansplaining*, and/or *Gaslighting* as discriminatory situations.

The incidence of these behaviors can be observed in various areas; however, in a predominantly male environment, the speech of women and their expressions and opinions are reduced. The higher male incidence in the scientific sector probably influences the representation of sexism reported by researchers in this study, although changes are expected as discussions on gender in science advance [5, 8]. For Fairchild et al. [26], situations that diminish women in their work environment transmit hostility, objectification, and exclusion.

The higher incidence of *Manterrupting* among the situations observed in this study shows the importance of a vigilant academic environment towards this behavior. Snyder [27], for example, conducted an informal study within her own workplace to test whether women were actually more interrupted than men at technology meetings. She identified that 90% of her colleagues are men, and they interrupt twice as much as women (every 2 min for 15 h of meeting time) and are three times more likely to interrupt women than other men.

Like *Manterrupting*, the other situations reported by women in this study may be considered subtle forms of sexism that over time accumulate and can lead to asymmetrical representation between men and women in work environments [28]. These situations do not occur separately: about 40% of participants quoted have already undergone more than one of the situations.

In a more detailed and qualitative manner, the Discourse of the Collective Subject Analysis expressed these situations of sexism and discrimination due to gender in the academic environment, revealing the presence of reports on (1) cognitive inferiority, (2) moral harassment, (3) physical inferiority, and (4) prejudice with motherhood (Table 2). The first report addresses the underestimation of female intellectual capacity in scientific activities, strictly related to *Mansplaining*. *Mansplaining* is strictly related to *Manterrupting*, and in this situation, a man thinks he knows more about a topic than a woman and often interrupts to show that he knows more, ultimately disregarding what has been said.

**Table 2:** Analysis of the Discourse of the Collective Subject in relation to situations of sexism or discrimination due to gender in the academic environment

Key expressions	Main idea
“There is an underestimation of my abilities by men in academics. I felt that my opinion was not considered, men associated my opinion with the fact that I was a woman, ignoring all the study and knowledge that I could have.”	Cognitive inferiority
“A fellow researcher exposed me to embarrassing and uncomfortable situations due to chauvinistic remarks, when I began to question his attitudes, he said that or I stayed or he stayed at work.”	Harassment
“I was replaced by a man on the team because they thought a woman could not withstand being in the field, they think women are more fragile, so they want to restrict our presence in situations that bring physical dangers or efforts.”	Physical inferiority
“I have stopped being selected to fill positions/vacancies as a researcher/professional because I am a mother. I was harassed on a selection committee where one of the examiners questioned if I had a small child to take to school.”	Prejudice with motherhood

The underestimation of the female gender in scientific activities is discussed by Gauthier et al. [28], who says that this vision begins during the school phase, in which boys already have a socially constructed frame of the scientific and physical incapacity of the girls. Additionally, the authors suggest that teachers often reinforce these stereotypes of girls as less skilled in math and science, validating the view of inferiority between the sexes [28]. Thus, this concept of intellectual inferiority permeates throughout the ontogenetic development of adolescents and is expressed in several spheres in their lives, such as their professional life.

In many cases, the expressions of sexism as *Mansplaining* or *Manterrupting* become more powerful. This can be observed when the Collective Discourse points to an incidence of moral and physical inferiority, demonstrating the exposure to embarrassing and uncomfortable situations in the work environment, as well as judging the interviewee’s inability to participate in activities that require physical effort. According to Clancy et al. [29], field activities, for example, are essential components of life, earth, and social sciences, and a lot of research is generated in the field context. However, the authors point out that various types of harassment occur during these activities and women are the main victims. The authors characterize these abuses as a form of undervaluing women within these activities by their colleagues or professors, as well as situations of embarrassment and verbal and sexual harassment.

Another important situation reported by the participants was the prejudice with motherhood, demonstrating a diminishing neglect of women just for the simple fact of being a mother. Williams and Ceci [30] found that women deal with more challenges in the academic world than men, yet when they choose to be mothers, they face even greater problems. For these authors, children change the professional scenario for women; however, this does not have the same effect in the professional life of men and is generally one-sided.

**Fig. 3:**
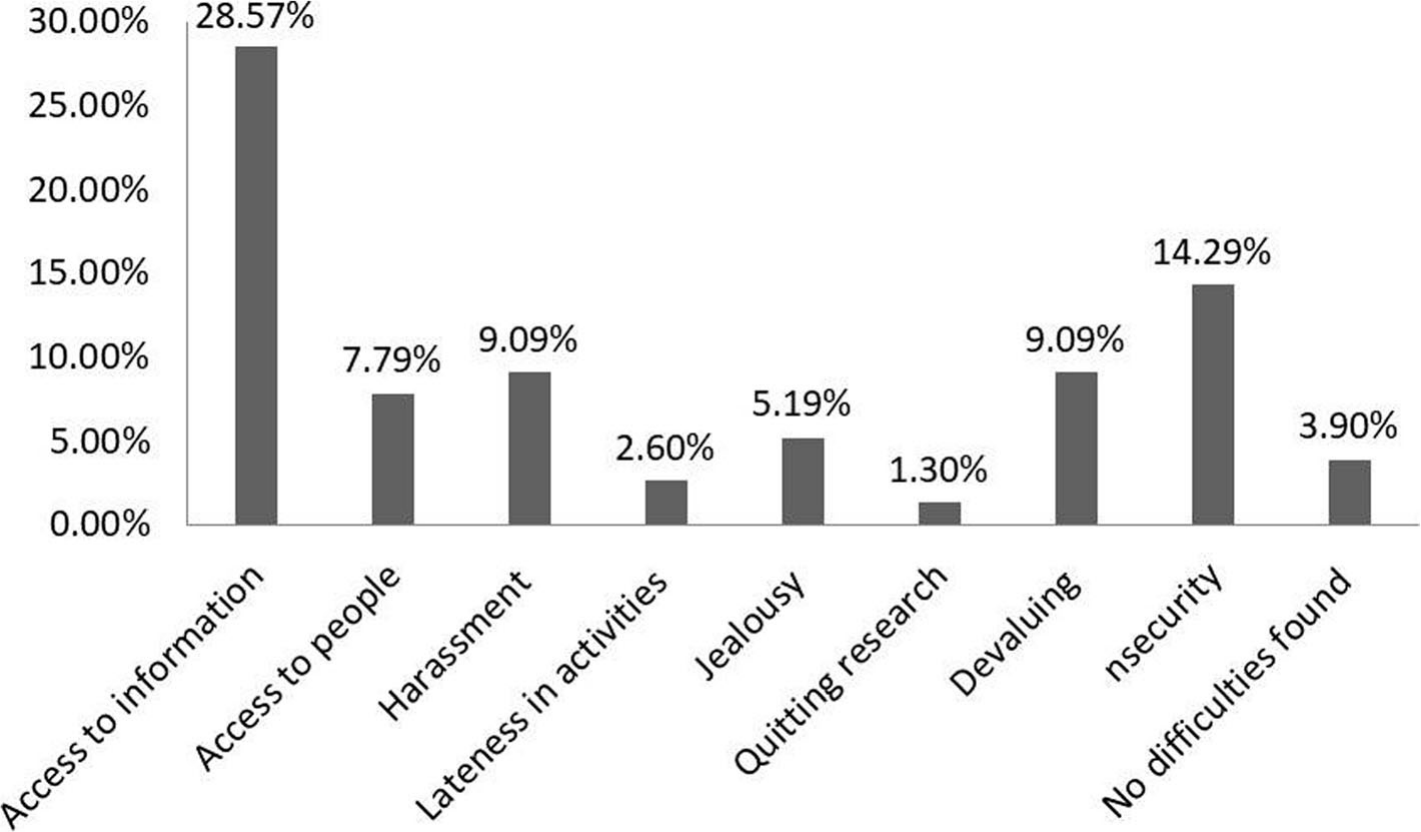
Perception of the Brazilian scientists interviewed in relation to disadvantages in ethnobiological research because they were women (*n* = 77 female ethnobiologists)

When researchers were questioned about whether or not they felt prejudiced in fieldwork because they were women, 62.3% said yes. This result demonstrates the disadvantages females face in the process of collecting ethnobiological data, which can be detrimental to several factors, such as access to information, access to people, harassment, and devaluing, among others (Fig. 3). This is because many times the research participants do not trust and/or feel comfortable giving information to women about their cultural practices related to natural resources, a factor that may be associated with the patriarchy and the recurrent sexism in these communities, such as the response to harassment by female researchers. Thus, it is important that research groups are composed by both women and men, in order to reduce cultural restrictions and other ethical and methodological problems, as reported by Pfeiffer and Butz [31].

Other disadvantages reinforce the argument of sexism, since the participants said that they felt insecure in the field. This insecurity is a reflection of situations of sexism, which is further aggravated when considering aspects that go beyond the academic sphere: many studies with peoples and traditional communities are inserted in contexts that, historically, reveal serious situations of insecurity. An example is the endemic conflicts over access to and ownership of land, as well as increasing threats to human rights and people’s lives [32]. When it comes to female researchers in the field, among other disadvantages were delays in research and even dropping out at the conclusion of the study, probably due to the factors listed above, which may decrease female representation in ethnobiology, as well as other areas of study (see [7, 12]).

In the collective discourse analysis, we observed some detailed reports on the disadvantages of being a woman in ethnobiological fieldwork (Table 3). The researchers expressed cases of insecurity, as well as a lack of autonomy in the community, research, and physical inferiority and a lack of data reliability. According to Lisboa [33], these facts are also commonly reported among women professionals in the fields of biology, geography, ecology, engineering, veterinary medicine, and other professionals who work with the environment and conduct field research in Brazil. In many situations, women go out to work at night for monitoring wildlife, for example, and can be startled because they live in an insecure, unequal, sexist, and paternalistic society. Growing movements in the country and in the world, such as *Parent in Science* and *Women in Science*, have been encouraging, empowering, and openly discussing the implications of being a woman and a professional in field activities. Thus, in a dialog between researchers of these groups, four major issues were raised about the obstacles faced by women in the field: (a) “company”—it is advisable for women during fieldwork to be accompanied by a field assistant, forest guide, or local guide because on certain occasions, the simple presence of a male is enough to inhibit unwanted male behaviors from other men; (b) extra costs—there is a need to allocate financial resources to pay for these field assistants (“men”), so this cost generates a decrease in resources that could be allocated to other purposes, such as acquisition of equipment, fuel, or more days in the field; (c) fear—the stress of insecurity hampers work, especially in areas of conflict, remote areas, and indigenous communities with different ethnicities and cultures, and going through fieldwork with the constant fear of being attacked by men is discouraging; and (d) trivialization of violence—in many regions of the country, the issue of violence against women is trivialized, including by women themselves. The effects of these adversities end up surpassing the fieldwork itself, which is reflected in scientific results, as well as its publication [34]. Within intercultural contexts, the social immersion that must be done to conduct research often for long periods leads the researcher to a series of risks that were not idealized in the construction of her research project.

**Table 3:** Analysis of the Discourse of the Collective Subject in relation to situations in which the researcher felt disadvantaged or prejudiced because she was a woman during fieldwork in ethnobiology/ethnoecology

Key expressions	Main idea
“Situations where it was just me as a woman and I felt insecure or scared… Once while I was working, I suffered a persecution… I do not recommend being alone in field work.”	Insecurity
“In the communities where I worked women do not make decisions…”	Lack of female autonomy in the community
“It is impossible to accompany a fishing expedition that is carried out solely by men… You cannot be on board to see interactions of fishing with dolphins.”	Lack of researcher autonomy
“In activities of greater physical effort, we always depended on a man to help us… I had problems with this.”	Physical inferiority
“One of the participants refused to tell me the use of a plant, but said quietly in the ear of my colleague who was a man…”	Data reliability

**Fig. 4:**
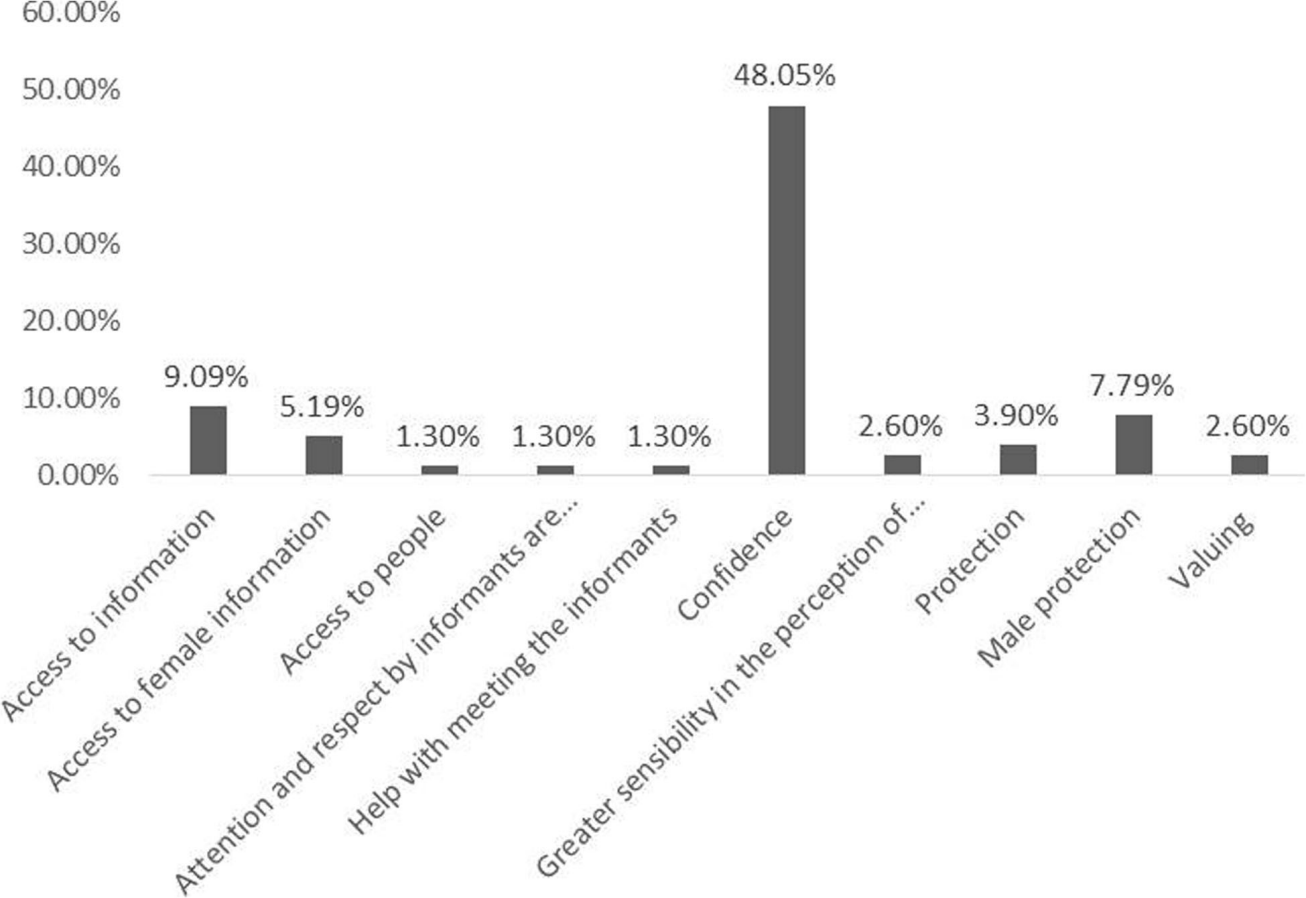
Perception of the advantages of being a woman in ethnobiological research in Brazil based on 77 interviews with ethnobiology scientists

When the researchers were asked if there are perceived benefits of being a woman, 60.0% responded yes. Among the benefits were access to information, access to people, and respect for the participants, among others (Fig. 4). The result demonstrates some antagonism regarding disadvantages that the researchers reported above, because some advantages of being a woman in Brazilian ethnobiological research was also recognized. These results are not antagonic and rather show different characteristics of complex contexts. It is obvious that when dealing with people, researchers were facing a diversity of behaviors within the same community. The perceived benefits can be related to empathy or a good rapport and can leverage advantages in the field related to gender. These advantages may be associated with the recognition of women as a fragile sex by a patriarchal community and women therefore require help and protection from interviewees [35]. The perception of the benefits of being a woman may be associated with the increasing female empowerment that, in addition to perceiving and discussing gender asymmetries in science, changes the perspective of aspects that were previously seen as fragilities and transforming them as a potential way of counterbalancing socially constructing gender differences [8].

**Fig. 5:**
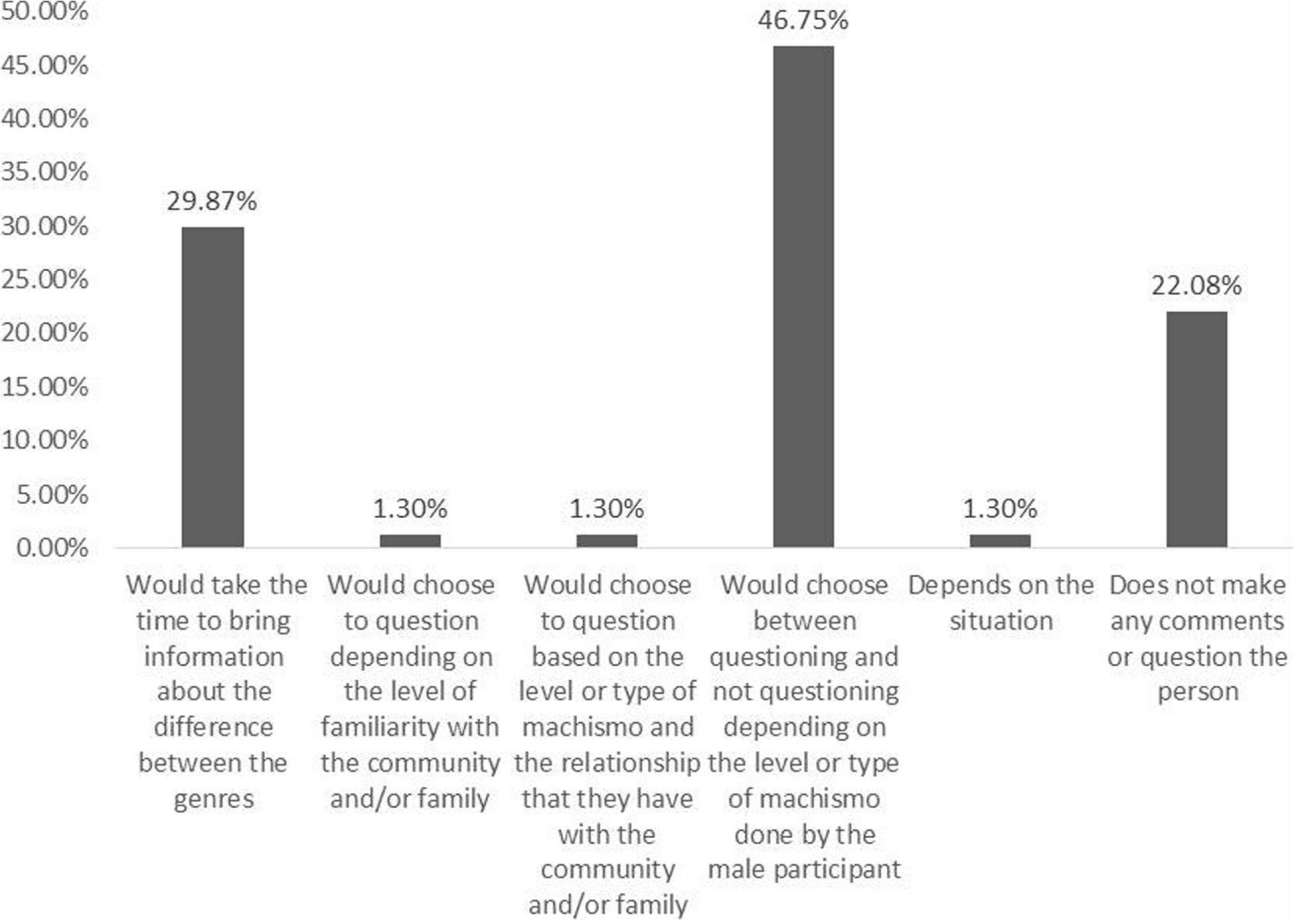
Reaction of the 77 participants when suffering sexism in the communities studied in Brazil

Regarding the researcher’s position in the face of explicit sexism suffered during fieldwork, we found that less than one third of the participants (29.9%) would take advantage of the moment to question and position themselves, while another part of the participants would only position themselves depending on the type or degree of sexism (46.7%) (Fig. 5). This behavior was already expected, since in ethnobiology researchers are keen to have a good relationship with their collaborators to ensure a good quality of data collection, always seeking to establish a rapport [19]. However, it is important to reflect to what extent neutrality in certain situations of sexism should prevail in defense of science. Many of these situations may jeopardize the safety and even the lives of researchers and/or local participants of the research (Table 4).

**Table 4:** Analysis of the Discourse of the Collective Subject in relation to situations of sexism or discrimination due to gender in ethnobiology/ethnoecology fieldwork

Key expressions	Main idea
“A group of men from the community harassed us whenever we passed a certain bar in the community...”	Sexual harassment
“Some male respondents had the opinion that fieldwork should not be developed by female researchers… they commented that I was weak to keep walking.”	Physical inferiority
“The husband came outside and said he could not answer me because his wife was not home…”	Data collection restriction

The Discourse of the Collective Subject details the situations of sexism faced by researchers in the field (Table 4). Among the reports were sexual harassment, physical inferiority, and restriction in data collection, as was reported when questioned about gender disadvantages in the field.

**Fig. 6:**
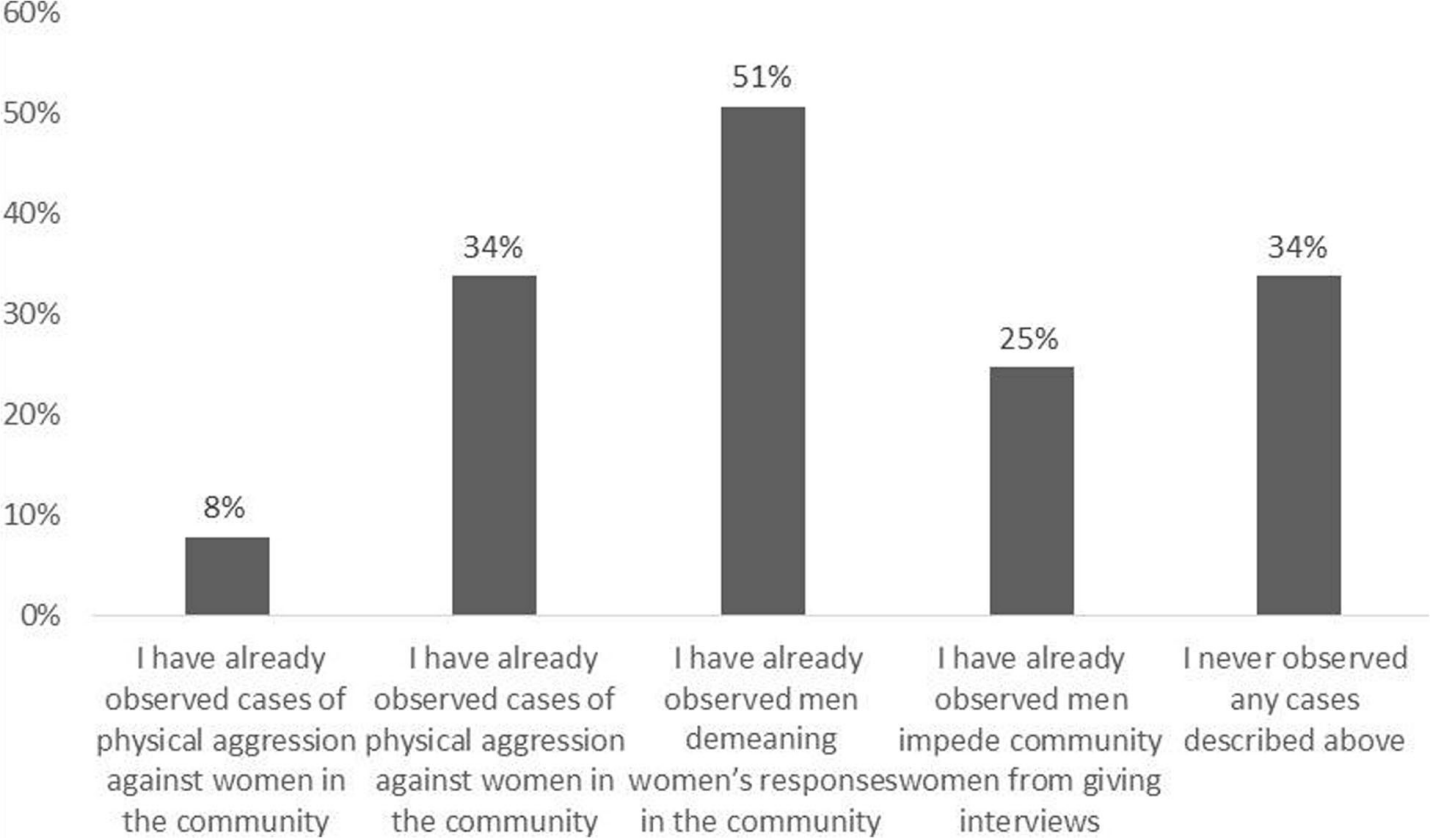
Cases of sexism observed in the communities studied by ethnobiologists in Brazil (*n* = 77 female ethnobiologists)

When the participants were questioned if there were cases of male chauvinism directed at women in the studied communities, the results pointed to several situations, such as men disregarding the responses given by women in the community (51.0%) and psychological aggression (34.0%), among others (8.0%), including physical aggression towards women (Fig. 6). These results show that we are facing a delicate discussion that involves the researcher positioning themselves, regardless of gender, in relation to individual and collective ethical aspects. According to the code of ethics of the International Society for Ethnobiology, “The fundamental value underlying the Code of Ethics is the concept of mindfulness - a continual willingness to evaluate one’s own understandings, actions, and responsibilities to others” [36]. In cases of violence witnessed in the field, this implies recognizing, as researchers, a tenuous line of our responsibilities to others in the collective (community) or towards others individually (women; Table 5).

**Table 5:** Analysis of the Discourse of the Collective Subject in relation to situations in which the researcher witnessed sexism suffered by women of the communities/groups with whom they worked

Key expressions	Main idea
“Women who picked up their drunken husbands was very common… I have already observed various forms of sexism and aggression against women in the daily life of the communities.”	Violence against women
“Men openly cheat at parties with their neighbors.”	Marital problems
“Women working in excess, looking after children, home, farming, and fishing.”	Excessive exploitation of women
“Most often when the woman spoke she was scolded… When interviewing the couple, the man always responded and sometimes he would call on the woman.”	Lack of female autonomy in the community

## Conclusions

Although ethnobiology approaches research with cognitive, perceptual, and cosmological understanding about natural resources between genders, it is necessary to go further, since these aspects range from methodological aspects to those that permeate the practices of researchers in this area of study. Thus, it is important to consider that because it is a science that deals with people and knowledge of people, it is necessary to assume that for many situations, we will be dealing with information that is gender sensitive, which can range from types of questions and hypotheses to methodological aspects and ethical practices that influence ethnobiological practice and data reliability [31]. However, the stereotyped beliefs of gender-sensitive situations, such as benevolent sexism [37], may limit women’s exposure to challenges and create consequences of under-representation of women in leadership positions.

Additionally, this study shows results that express advantages and disadvantages of ethnobiological practice in some situations related to the female gender. It is important to emphasize the disadvantages and the general trend in the field of sciences, where women have reached the top of the career to a lesser extent than men, which is shown by the bias of senior authors in scientific publications. However, the absence of bias among first authors indicates a positive change in female participation in ethnobiology. Even though we have positive scenarios regarding female participation as the first authors in ethnobiological studies, it is worth remembering the arguments presented by Pietri et al. [1]. These arguments contend that informing women about gender bias in different areas of science increases their identification with a female scientist, and such identification can be an important intervention even to protect women from harmful consequences associated with bias, such as reducing confidence.

The imbalance produced by the absence of women in science has been discussed in several countries. We note that there is a document from the US National Academy of Science and Engineering, which indicates that “a greater presence of women in the world of science and technology is essential for scientific excellence and also for the country’s economic development.” The European Research Area Vision also set a clear goal for 2030—“Half of all scientists and policy makers in all disciplines and at all levels of scientific system will be women.” Thus, the European institutions are developing mandates for gender mainstreaming as established in the Treaty of Amsterdam in the field of science, whose principle of gender mainstreaming was adopted by the United Nations at the World Conference on Women in Beijing (1995) [38].

Lastly, it is important to remember the asymmetries related to gender and simplified when considering only binary situations (male/female) [8] are just one of the asymmetries that permeate scientific activities in ethnobiology and other sciences. Western science was built not only with clear gender biases [5], but also with an ethnic bias, and ethnobiology has also an important role to question these asymmetries.

## Data Availability

Please contact the author for data requests.
